# Remodeling Factors, Transcription Factors and Angiogenetic Factors in Cholesteatoma in Ontogenetic Aspect

**DOI:** 10.22038/IJORL.2021.53716.2842

**Published:** 2022-03

**Authors:** Kristaps Dambergs, Gunta Sumeraga, Māra Pilmane

**Affiliations:** 1 *Department of Otorhinolaryngology, Riga Stradins University, * *16 Dzirciema Street, LV-1007* *, Riga, Latvia. *; 2 *Institute of Anatomy and Anthropology, Riga Stradins University, Kronvalda Boulevard 9, LV–1010 Riga, Latvia.*

**Keywords:** Cholesteatoma, Transcription factor, Metalloproteases, Vascular endothelial growth factor

## Abstract

**Introduction:**

The main goal of our study was to describe the transcription factor (NF-κβ), angiogenetic factor (VEGF), and remodeling markers (MMP-9 and TIMP-4) of the cholesteatoma tissue compared to control skin tissue. There are still uncertainties how transcription, angiogenetic and remodeling factors affect the cholesteatoma course.

**Materials and Methods:**

Eight cholesteatoma tissue specimens were retrieved from children, seven – from adults, seven skin controls – from cadavers. Obtained material immunohistochemically were stained for NF-κβ, MMP-9, TIMP-4, VEGF. Non-parametric statistic methods were used.

**Results:**

A statistically significant higher numbers of NF-κβ and TIMP-4 immunoreactive cells in the cholesteatoma compared to control group. A very strong positive correlation between MMP-9 and TIMP-4 was seen in the patient group. A strong positive correlation - between MMP-9 in matrix and MMP-9, VEGF in perimatrix, between TIMP-4 in matrix and TIMP-4 in perimatrix, NF-κβ in the matrix and VEGF; between TIMP-4 in perimatrix and NF-κβ in the matrix.

**Conclusions:**

Correlation between MMP-9 and TIMP-4 suggests that TIMP-4 in cholesteatoma tissue intercorrelates to MMP-9. TIMP-4 likely regulates the development of cholesteatoma. Disbalance between MMPs and TIMPs affects NF-κβ and causes uncontrolled cell proliferation and immune response in this tumor. There is a lack of VEGF strong expression in cholesteatoma perimatrix.

## Introduction

Acquired cholesteatoma is a noncancerous keratinized squamous epithelium growth found mostly in the middle ear (1). Different cell factors are responsible for development and progression of the cholesteatoma. We suggest that transcription, angiogenetic, and remodeling factors might be important in the course of cholesteatoma pathogenesis. 

Transcription factor nuclear factor-kappa beta (NF-κβ) is found in all nucleated cell types (2). NF-κβ regulates cytokine production and inflammatory immune response. 

NF-κβ is important in inflammation, cell proliferation and differentiation, immune response, carcinogenesis, and protection against apoptosis (3,4).

NF-κβ can be activated after various stimuli, which induce the phosphorylation of I*κ*βs (inhibitor kappa beta) (5). NF-κβ is highly activated in cholesteatoma compared to unchanged adjacent tissue, which is showed by Li et al. (6) It is believed that activated NF-κβ might cause cellular proliferation and provide protection from apoptosis and, therefore, is one of the factors for the development of cholesteatoma (5). Several authors have reported overexpression of NF-κβ in cholesteatoma tissue in comparison to the control group (5-8). 

Vascular endothelial growth factor (VEGF) was first found in endothelial cells. It is known that VEGF is found in various non-endothelial cells, like macrophages (9), keratinocytes (10) and tumor cells (11). Angiogenesis is one of the critical signs in inflammatory granulation tissue, which is common in cholesteatoma (12). Mostly angiogenesis in cholesteatoma is seen in the perimatrix, which is subepithelial connective tissue (13). It is proven that angiogenesis helps the growth of cholesteatoma tissue, which is similar in various tumor tissue (14). In chronic middle ear infection, VEGF is known to be one of the most potent angiogenetic factors (15-17). It is responsible for proliferation of endotheliocytes (18). Fukudome et al. (2013) have noted that keratinocytes in the cholesteatoma matrix might produce the angiogenetic factors, which are then delivered in the perimatrix to induce angiogenesis in a paracrine manner (19).

Matrix metalloproteinases (MMPs) can cause the degradation of extracellular matrix (ECM), which is essential in oncologic and inflammatory processes, that is why several authors research the interaction of remodeling factors in cholesteatoma (20,21). MMPs proteolytic activity is regulated by tissue inhibitors of metalloproteinases (TIMPs). Disbalance between MMPs and TIMPs in cholesteatoma compared to unchanged tissue might be important in processes happening in cholesteatoma patients (22,23). MMP-9 in cholesteatoma tissue is explicitly seen in areas with inflammatory cell infiltration, and also is seen in basal and suprabasal levels of the matrix (21-23). 

There are some uncertainties about whether MMP-9 is upregulated in cholesteatoma compared to unchanged skin. Some authors, like Holt et al. (24), Schmidt et al. (21), Banerjee et al. (25) have proved a correlation between the aggressiveness of cholesteatoma and increased levels of MMP-9 and MMP-2. In opposition, Rezende et al. (26), in their study, showed no upregulation of MMP-9 in cholesteatoma. Olszewska et al. (27) and Juhasz et al. (28) presented that MMP-9 is overexpressed in cholesteatoma perimatrix compared to unchanged skin tissue, and that is associated with bone resorption. Also, TIMP-1, which is an MMP-9 specific inhibitor, was found to be upregulated in cholesteatoma, and it indicates that there is a dynamic balance between these two remodeling factors in cholesteatoma (27). TIMP-4 has the most potent inhibitory action against MMP-26 (29-31). Also, there is a direct or indirect action against MMP-2 and MMP-9 induction, but the mechanism is not entirely understood (32). Shynlova et al. (33) suggest that TIMP-4 has a role in ECM protection from proteolysis. 

Commonly, there are limited publications on TIMP-4 effect on the regulation of ECM proteolysis (34). Therefore, there are no scientific studies and information related to TIMP-4 and cholesteatoma. Therefore, the aim of our work was to describe the distribution of transcription factor (NF-κβ), angiogenetic factor (VEGF), and remodeling markers (MMP-9 and TIMP-4) of the cholesteatoma.

## Materials and Methods

*Cholesteatoma and control tissue*

Cholesteatoma specimens were retrieved during cholesteatoma surgery in Children’s Clinical University Hospital and P. Stradins Clinical University Hospital, but the morphological analysis and immunochemical staining of the tissue were conducted at the Department of Morphology of the Riga Stradins University, Latvia. Eight cholesteatoma tissue samples were obtained from children, five males, three females (age 9–17 years, mean age 14.87 years). Seven cholesteatoma specimens were obtained from adults, two male, five female patients (age 23–75 years, mean age 42.71 years). Seven deep external meatal skin controls were obtained from cadavers (3 female, 4 males, age ranged from 40 – 75 years) in a collection of Institute of Anatomy and Anthropology. Ethical Committee of Riga Stradiņš University approved this study (05.09.2019; Nr.6-2/7/4). All of the patients or patient parents gave their informed consent to participate in the study. The nature of the study had been fully explained to the patients and patient parents. Inclusion criteria for patient group were: (1) patients with diagnosis of acquired middle ear cholesteatoma; (2) obtained material was sufficient to make immunohistochemical analysis; (3) presence of matrix and perimatrix in the cholesteatoma in immunohistochemical slides. Exclusion criteria were: (1) diagnosis of congenital cholesteatoma; (2) inadequate sample material after immunohistochemical analysis. In control group inclusion criteria were: (1) enough material for immunohistochemical evaluation; (2) no middle ear surgeries in patient history. Exclusion criteria were: (1) insufficient material after performing immunohistochemical analysis – missing epithelium and/or connective tissue; (2) known skin diseases, if mentioned in patient file. 

***Immunohistochemical Analysis***

Tissues were fixed in a mixture of 2% formaldehyde and 0.2% picric acid in 0.1 M phosphate buffer (pH 7.2). Afterward, they were rinsed in Tyrode buffer (content: NaCl, KCl, CaCl2_2H2O, MgCl2_6H2O, NaHCO3, NaH2PO4_H2O, glucose) containing 10% saccharose for 12 h and then embedded into the paraffin. Thin sections (3 µm) were cut, which were then stained with haematoxylin and eosin for routine morphological evaluation. The Biotin-Streptavidin biochemical method was used for immunohistochemistry (IMH) to detect: VEGF (orb191500, rabbit, polyclonal, working dilution 1:100, Biorbyt Ltd., United Kingdom), MMP-9 (sc-10737, rabbit, working dilution 1:100, Santa Cruz Biotechnology, Inc., Santa Cruz, CA, USA), TIMP-4 at 1:100 (sc-30076, rabbit, working dilution 1:100, Santa Cruz Biotechnology, Inc., Santa Cruz, CA, USA), and NFkB-105 (obtained from rabbit, 1:100 dilution, Abcam, UK). The slides were analysed by light microscopy by two independent morphologists using semi-quantitative method. 

The results were evaluated by grading the appearance of positively stained cells in the visual field (35). Structures in the visual field were labeled as follows: 0 = no positive structures, 0/+ = occasional positive structures, + = few positive structures, +/++ = few to a moderate number of positive structures, ++ = moderate number of positive structures, ++/+++ = moderate to numerous positive structures, +++ = numerous positive structures, +++/++++ = numerous to abundant structures, ++++ = an abundance of positive structures in the visual field (35). Leica DC 300F digital camera and image processing and analysis software Image-Pro Plus (Media Cybernetics, Inc., Rockville, MD, USA) were used for a visual illustration.

***Statistical Analysis***

The data processing was performed with SPSS software, version 23.0 (IBM Company, Chicago, IL, USA). We used Spearman’s rank correlation coefficient to analyze correlations between different cell factors, where r = 0–0.2 was assumed as a very weak correlation, r = 0.2–0.4 a weak correlation, r = 0.4–0.6 a moderate correlation, r = 0.6–0.8 a strong correlation, and r = 0.8–1.0 a very strong correlation. The Mann-Whitney U test was used to analyze the difference between control and patient group. Two-tailed p values < 0.05 were considered statistically significant for all statistical calculations. 

## Results

***Findings of routine histological analysis***

**Fig 1:**
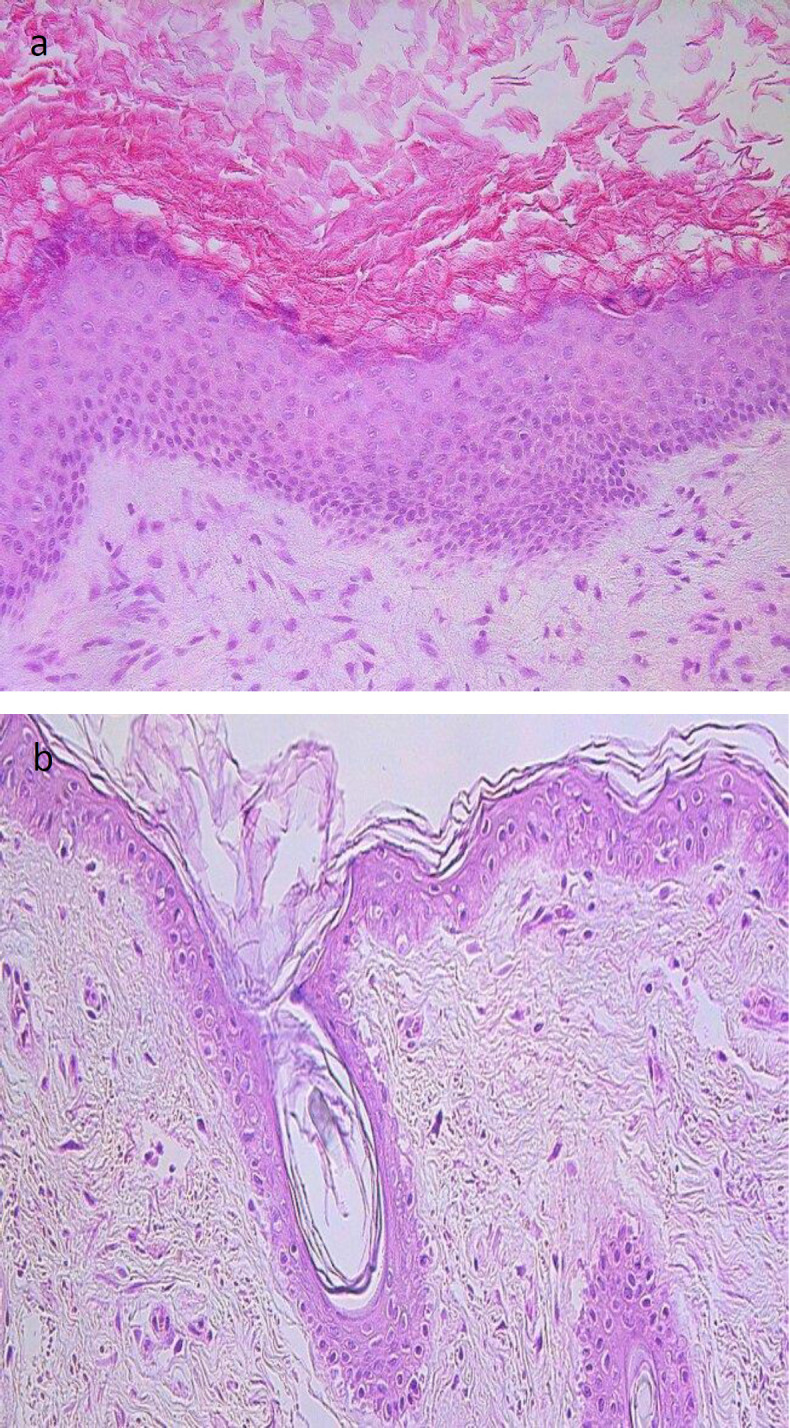
Micrographs of cholesteatoma and control skin tissue. (**a**) Cholesteatoma in a 58 years old patient. Cystic layer mostly consists of desquamated, anucleate keratin mass, matrix with hyperproliferative stratified squamous epithelium and perimatrix subepithelial connective tissue with some consisting of inflammatory cells. Haematoxylin and eosin, X 200; (**b**) Control material demonstrates unchanged skin from the external ear canal. Haematoxylin and eosin, X 200

Cholesteatoma tissue showed a cystic layer with anucleate keratinocytes, matrix – hyperproliferation of epitheliocytes were seen, and perimatrix displayed infiltration of inflammatory cells (Figure 1a). External ear canal skin (control group) displayed unchanged stratified squamous epithelium and connective tissue with absence of inflammation (Figure 1b). Tissue was stained with haematoxylin and eosin.

***Findings of immunochemistry and statistical analysis of*** ***NF-κ*** ***β***

**Fig 2:**
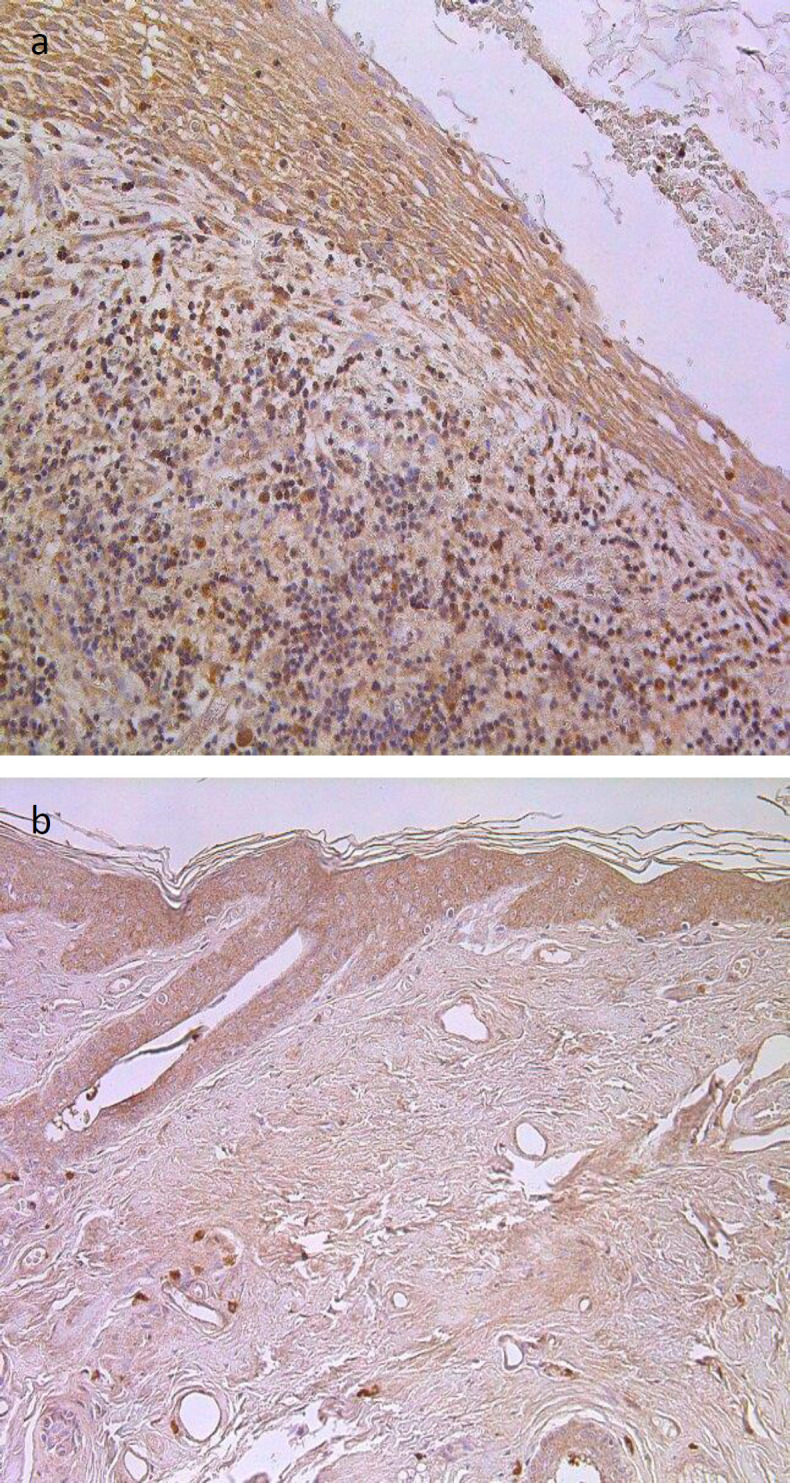
Immunohistochemical micrographs of cholesteatoma tissue and control group. (a) Numerous NF-κβ positive cells in matrix and perimatrix of a 38 years old cholesteatoma patient, NF-κβ IHC, X 200; (b) Moderate NF-κβ positive cells in the epithelium and a few to moderate in the connective tissue of a control skin sample, NF-κβ IHC, X 200

Numbers of NF-κβ-containing cells in the cholesteatoma matrix and perimatrix versus control group epithelium and connective tissue showed a statistically significant difference (Tables 1 and 2; Figures 2 a, b). We detected (on average) moderate NF-κβ containing cells in the matrix compared to occasional NF-κβ positive cells in skin epithelium (p=0.0004). In the perimatrix, we have seen few to moderate NF-κβ containing cells compared to an occasional in controls connective tissue (p=0.0003) (Tables 1 and 2, Figures 2a and b).

***Findings of immunochemistry and statistical analysis of*** ***VEGF***

**Fig 3:**
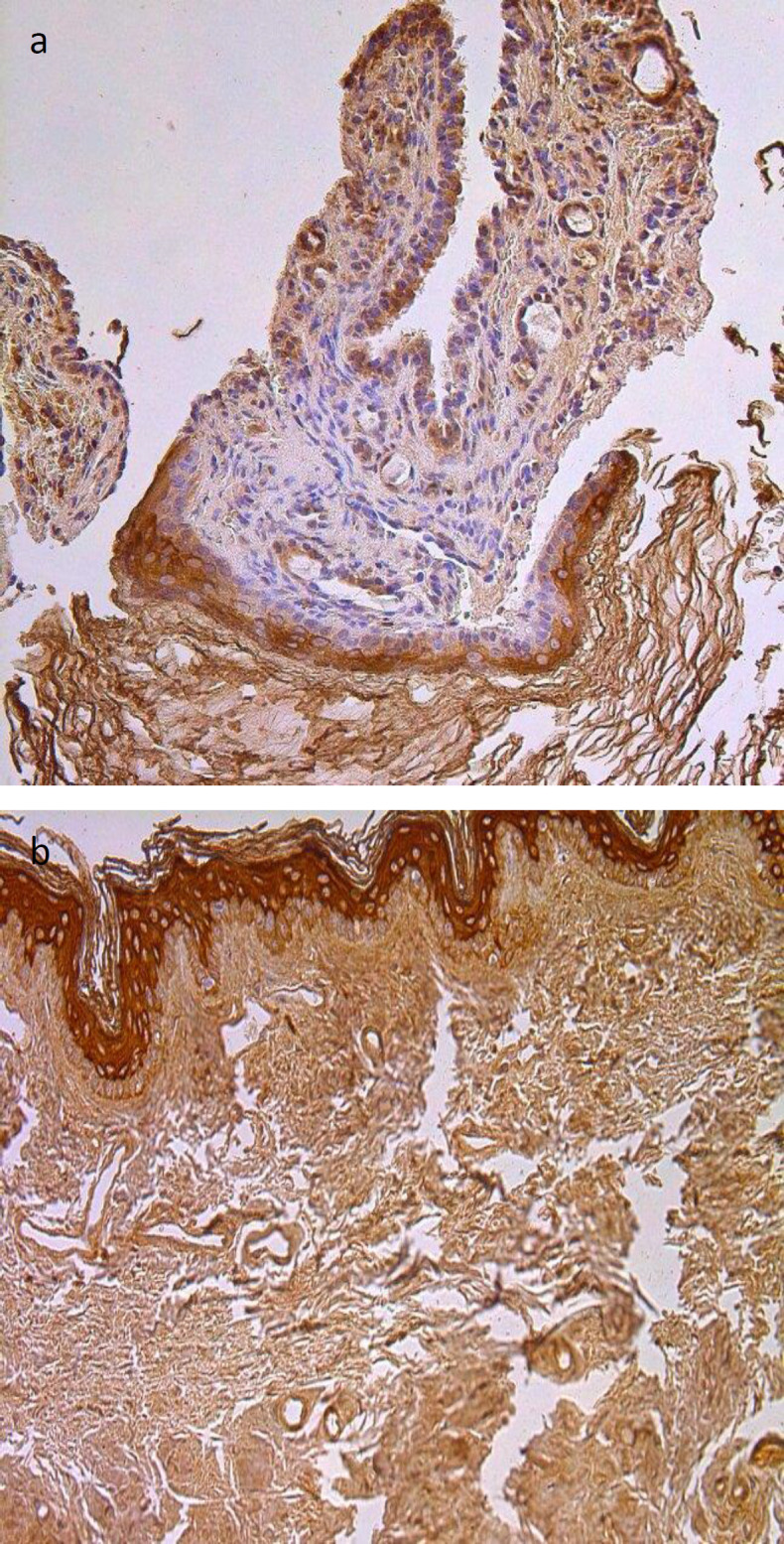
Immunohistochemical micrographs of cholesteatoma tissue and control group subjects. (a) Moderate VEGF positive endothelial cells of a 28 years old cholesteatoma patient, VEGF IHC, X 200; (b) A few to moderate VEGF positive endothelium cells of a control skin sample, VEGF IHC, X 200

No statistically significant difference was found between the amount of VEGF-containing endothelial cells in cholesteatoma perimatrix (on average a few) and control group connective tissue where we detected occasional VEGF positive endothelial cells (Table 1, Figures 3 a and b).

***Findings of immunochemistry and statistical analysis of*** ***MMP-9***

**Fig 4:**
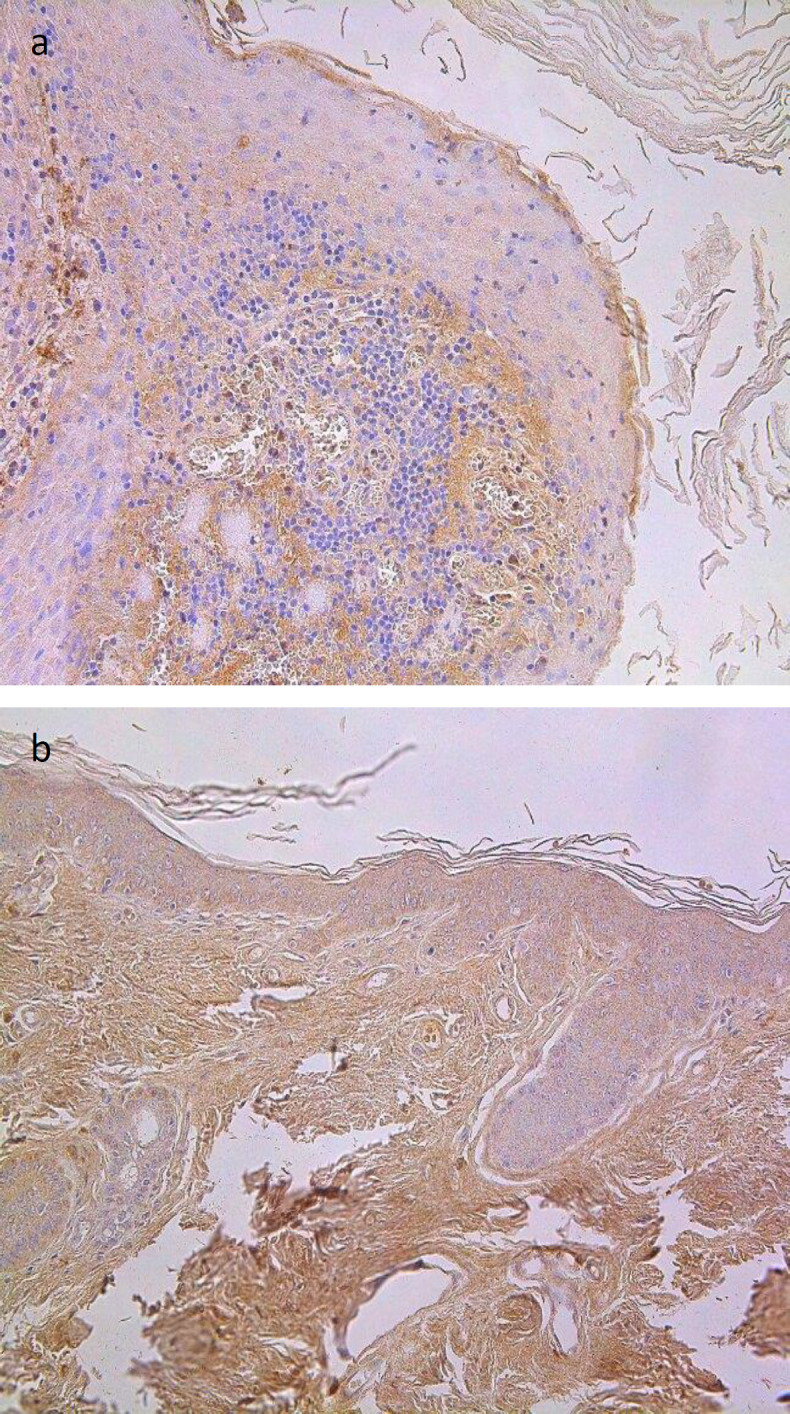
Immunohistochemical micrographs of cholesteatoma tissue and control group subjects. (a) A few to moderate MMP-9 positive cells in matrix and a few perimatrix of a 28 years old cholesteatoma patient, MMP-9 IHC, X 200; (b) Moderate MMP-9 positive cells in the epithelium and a few to moderate in the connective tissue of a control skin sample, MMP-9 IHC, X 200

Remodeling factor MMP-9 varied from occasional to moderate immunoreactive cells in the cholesteatoma matrix, the same amount of MMP-9 immunoreactive epitheliocytes was seen in the control group. MMP-9 immunoreactive cells in the cholesteatoma perimatrix varied from occasional to a few to moderate. The same amount of MMP-9 positive cells in the control group’s connective tissue (Table 1, Figures 4a and b). No statistically significant difference was seen between the groups.

***Findings of immunochemistry and statistical analysis of*** ***TIMP-4***

TIMP-4-containing epitheliocytes in cholesteatoma’s matrix marked a variance from a few to numerous to abundant, which was slightly more than in deep meatal skin epithelium, where it was from a few to moderate to numerous cells. However, no statistically significant difference was detected (Table 2). TIPM-4 immunoreactive cells in the perimatrix (moderate to numerous) showed a statistically significant difference from TIMP-4 positive cells in control connective tissue (on average, a few to moderate) (p=0.0085), (Tables 1 and 2, Figures 5a and b).

**Fig's 5 a, b:**
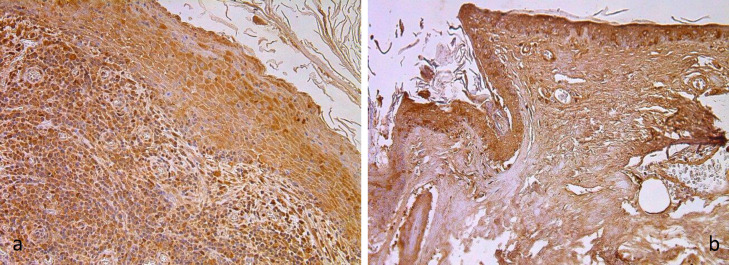
Immunohistochemical micrographs of cholesteatoma tissue and control group subjects. (a) Numerous TIMP-4 positive cells in matrix and perimatrix of a 28 years old cholesteatoma patient, TIMP-4 IHC, X 200; (b) Moderate to numerous TIMP-4 positive cells in the epithelium and moderate in the connective tissue of a control skin sample, TIMP-4 IHC, X 200

**Table 1:** Immunoreactivity of remodeling, transcription and angiogenetic factors in patient and control group

**N**	**Age**	**MMP-9** **M**	**MMP-9** **P**	**TIMP-4** **M**	**TIMP-4** **P** *	**NF-κ** **β** **M** *	**NF-κ** **β** **P** *	**VEGF** **P**
P1	9	00/+	0/+	+	+/++	0/+	0/+	0
P2	10	+	0/+	+++/++++	+++/++++	++/+++	+	++
P3	11	0/+	0/+	++/+++	++	++/+++	++	0/+
P4	13	++	+/++	+++/++++	+++	+++	++	++
P5	16	0/+	00/+	++/+++	++/+++	++	+	0
P6	16	++	+/++	+++/++++	+++	+++	+/++	++/+++
P7	16	++	0/+	+++/++++	++	++	+/++	++
P8	17	0	0	++	++	++	+/++	0/+
P9	23	+	0/+	++/+++	++/+++	++	++	0/+
P10	28	+	+	+++	+++	++	++	++
P11	31	++	+	+++/++++	+++	++/+++	++	0/+
P12	38	0/+	0/+	++/+++	++/+++	+++	+++	0
P13	46	0/+	0/+	+/++	+/++	+/++	+	0
P14	58	00/+	0/+	+/++	+++	++	+++	0/+
P15	75	0/+	+	++/+++	++	++	++	0
AVG	26,46	+	0/++	++/+++	++/+++	++	+/++(+)	+
N	-	MMP-9 E	MMP-9 CT	TIMP-4 E	TIMP-4 CT	NF-κβ E	NF-κβ CT	VEGF CT
C1	-	+	0/+	++	+	0	00/+	+
C2	-	+/++	+	++/+++	++	0/+	0/+	+
C3	-	0/+	0/+	+/++	+/++	0	0	0/+
C4	-	++	0/++	++/+++	++	+	0/+	+/++
C5	-	++	+/++	+++	++	++	+/++	+
C6	-	+	0/+	++/+++	++	+	0/+	0/+
**C7**	-	+	0/+	+	+	0/+	0	0
AVG	-	+	0/++	++	+/++	0/++	0/+	0/++

**Table 2:** Mann–Whitney U test revealing statistically significant differences in cell positive factors between cholesteatoma patients and controls

Tissue factors	Mann – Whitney U test	*P*-value
**Difference between TIMP-4 in perimatrix and connective tissue of the skin **	17	0.0085
**Difference between ** **NF-κ** **β** ** in cholesteatoma matrix and control tissue epithelium**	8.5	0,0004
**Difference between ** **NF-κ** **β** ** in cholesteatoma perimatrix and control connective tissue**	7	0,0003


Intercorrelations between factors using Spearman’s rank correlation in patient group and control group: Correlations between different cell factors in the patient group are seen in Table 3. Correlations between different cell factors in the control group are seen in Table 4.

**Table 3:** Correlations (Spearman Rho) between the relative numbers of different tissue factors in cholesteatoma group

Factor 1	Factor 2	R	*P *- value
A very strong positive correlation			
**MMP-9 in matrix**	TIMP-4 in matrix	0.91	0,000006
Strong positive correlation			
**MMP-9 in matrix**	MMP-9 in perimatrix	0.67	0.008
**MMP-9 in matrix**	VEGF in perimatrix	0.67	0.008
**TIMP-4 in matrix**	TIMP-4 in perimatrix	0.64	0.010
**TIMP-4 in matrix**	NF-κβ in matrix	0.69	0.006
**TIMP-4 in matrix**	VEGF in perimatrix	0.73	0.003
**TIMP-4 in perimatrix**	NF-κβ in matrix	0.63	0.010
**TIMP-4 in perimatrix**	VEGF in perimatrix	0.63	0.020
Moderate positive correlation			
**MMP-9 in matrix**	NF-κβ in matrix	0.53	0.045
**TIMP-4 in matrix**	MMP-9 in perimatrix	0.56	0.030

**Table 4:** Correlations (Spearman Rho) between the relative numbers of different tissue factors in control group

Factor 1	Factor 2	R	*p *– value
A very strong positive correlation			
**TIMP-4 in epithelium**	NF-κβ in connective tissue	0.99	0.002
**TIMP-4 in connective tissue**	NF-κβ in connective tissue	0.85	0.030
**NF-κ** **β in epithelium**	NF-κβ in connective tissue	0.83	0.040
**MMP-9 in epithelium**	NF-κβ in connective tissue	0.82	0.050

Abbreviations: MMP-9 – matrix metalloproteinase 9; TIMP-4 – tissue inhibitor of metalloproteinase-4; NF-κβ – nuclear factor kappa beta

## Discussion

Cholesteatoma can cause local osteolytic processes in the middle ear, which can be associated with various complications. Some authors, like Olszewska et al. (27) and Juhasz et al. (28). stated that it is related to MMP-9 overexpression in cholesteatoma in comparison to unchanged skin. However, our results do not show any statistically significant difference between patients and controls, which is similar to Rezende et al. (26), where they show no upregulation of MMP-9 in cholesteatoma tissue. However, we showed a statistically significant difference between TIMP-4 in cholesteatoma perimatrix and controlled connective tissue. Our data shows that TIMP-4 is overexpressed in cholesteatoma. Thus, we suggest that TIMP-4 is relevant in the activity of cholesteatoma in the middle ear. The main function of TIMPs is to inhibit MMPs, but they also have many other functions (36); for example, TIMP-4 has a positive correlation with tumor aggressiveness (37,38). 

A literature review does not show articles regarding TIMP-4 relations with cholesteatoma. However, relatively recent studies suggest that overexpression of TIMP-4 might cause more rapid cervical cancer growth in mice (39). These assumptions about TIMP-4 being a stimulant of tumorigenic activity, based on our results, lead us to believe that TIMP-4 is likely associated with the aggressiveness of cholesteatoma. 

Additionally, we found that TIMP-4 in the cholesteatoma matrix has a very strong positive correlation with MMP-9 and a moderate correlation between TIMP-4 in matrix and MMP-9 in perimatrix. We did not find similar correlations in the control group tissue. It suggests that TIMP-4 is interconnected to MMP-9 in cholesteatoma. Navratilova et al. (40) made an assumption that TIMP-4 might affect bone resorption by inhibiting MMP-9. As we proved intercorrelation between MMP-9 and TIMP-4 in cholesteatoma, we suggest that these remodeling factors might also influence bone remodulation in middle ear bone.

Several stimuli can activate NF-κβ, and it is an important transcriptional factor that can regulate inflammation, epithelial-mesenchymal transition, angiogenesis, and tumorigenesis (41). Some theories suggest that overexpressed NF-κβ can cause cell hyper-proliferation in cholesteatoma tissue compared to unchanged skin epithelium (7). 

Our data strongly support previously reported findings (5-8) showing increased NF-κβ presence in cholesteatoma tissue cells compared to skin. Our research presented a statistically significant difference between NF-κβ-containing cells in matrix and perimatrix in comparison to the control group skin epithelium and connective tissue. These authors showed the importance of NF-κβ in cholesteatoma. Also, we detected a strong positive correlation between NF-κβ (matrix) and TIMP-4 (matrix and perimatrix), as well as a moderate positive correlation between MMP-9 in the matrix, which was similar to the control group tissue. It might be suggested that the disbalance between MMPs and TIMPs in cholesteatoma remodeling factors can affect NF-κβ and cause uncontrolled cell proliferation and immune response. 

Since there are no studies about how TIMP-4 affects cholesteatoma and is only limited information of TIMP-4 role in the organism, we found that our data are supported by Lizarraga et al. (39) ^Error! Bookmark not defined.^ Who concluded that overexpression of TIMP-4 affects NF-κβ by regulating it and, therefore, exhibiting pro-tumorigenic activity in mice cervical cancer. 

Fukudome et al. (19) displayed the importance of VEGF in cholesteatoma angiogenesis, but, in their research, VEGF was presented as a part of a cascade that can induce angiogenesis in several ways. In our study, we primarily examined endothelial cells, and we did not find statistically significant difference in their nucleus between patients and controls. This suggests that VEGF is not overexpressed in already developed blood vessels. However, we cannot exclude the induction of angiogenesis through a different signal pathway. 

Even though neo-angiogenesis was more observed in cholesteatoma tissue compared to unchanged skin and it is linked to the aggressiveness of cholesteatoma (19), we did not find a statistically significant difference of VEGF immunoreactive endotheliocytes between the patient and control group. However, we found a strong positive correlation between VEGF, TIMP-4 and MMP-9, but no such associations were detected in the control group. Again, this might indicate that a disbalance balance between remodeling factors can cause angiogenesis. These data can be supported by Abu El-Asrar (42). These authors concluded that correlations between VEGF and MMP-9 and TIMP-4 were found more in the eye tissue for patients with proliferative diabetic retinopathy, which characterizes with increased neo-angiogenesis. 

However, we realise that the present study has some limitations. An additional quantification of tissue markers by standardised laboratory measurements (e.g., ELISA) would benefit the purely visual evaluation of immunohistochemistry-stained samples. Also, larger group size for children and adult patients would be beneficial, so it would be possible to compare these groups. Furthermore, we acknowledge that the relatively small control group and that the material is taken from cadavers might pose a limitation to the study. Ethical considerations, however, mandate the use of this relative control group. Finally, we think it would be valuable to research genes and gene proteins which could be responsible for benign tumour development in the middle ear. 

## Conclusion

A very strong correlation between MMP-9 and TIMP-4 suggests that TIMP-4 in cholesteatoma tissue intercorrelates to MMP-9.

A statistically significant difference between TIMP-4 in perimatrix and skin connective tissue indicates that TIMP-4 likely regulates the development of cholesteatoma. The overexpression of NF-κβ and its correlations with remodeling factors in cholesteatoma proves that disbalance between MMPs and TIMPs affects NF-κβ and causes uncontrolled cell proliferation and immune response in this tumor.There is a lack of strong VEGF expression in cholesteatoma perimatrix, indicating the absence of its importance in the pathogenesis of cholesteatoma.
